# Interpretatively automated identification of circulating tumor cells from human peripheral blood with high performance

**DOI:** 10.3389/fbioe.2023.1013107

**Published:** 2023-02-09

**Authors:** Xiaolei Li, Mingcan Chen, Jingjing Xu, Dihang Wu, Mengxue Ye, Chi Wang, Wanyu Liu

**Affiliations:** ^1^ Sino-European School of Technology of Shanghai University, Shanghai University, Shanghai, China; ^2^ School of Mechatronic Engineering and Automation, Shanghai University, Shanghai, China

**Keywords:** circulating tumor cells, deep learning, single-shot multibox detector, interpretative analysis, precise identification

## Abstract

The detection and analysis of circulating tumor cells (CTCs) would be of aid in a precise cancer diagnosis and an efficient prognosis assessment. However, traditional methods that rely heavily on the isolation of CTCs based on their physical or biological features suffer from intensive labor, thus being unsuitable for rapid detection. Furthermore, currently available intelligent methods are short of interpretability, which creates a lot of uncertainty during diagnosis. Therefore, we propose here an automated method that takes advantage of bright-field microscopic images with high resolution, so as to take an insight into cell patterns. Specifically, the precise identification of CTCs was achieved by using an optimized single-shot multi-box detector (SSD)–based neural network with integrated attention mechanism and feature fusion modules. Compared to the conventional SSD system, our method exhibited a superior detection performance with the recall rate of 92.2%, and the maximum average precision (AP) value of 97.9%. To note, the optimal SSD-based neural network was combined with advanced visualization technology, i.e., the gradient-weighted class activation mapping (Grad-CAM) for model interpretation, and the t-distributed stochastic neighbor embedding (T-SNE) for data visualization. Our work demonstrates for the first time the outstanding performance of SSD-based neural network for CTCs identification in human peripheral blood environment, showing great potential for the early detection and continuous monitoring of cancer progression.

## 1 Introduction

According to a report from the World Health Organization (WHO), lung cancer mortalities had the highest morbidity and mortality worldwide among all malignancies in 2020 (Wild et al., 2020; Gutierrez-Sainz et al., 2021). As more than 70% of lung cancer patients have no symptoms in the early stage, it is usually too late to cure when realized. And more, the most commonly used imaging techniques for large-scale objects (such as computed tomography, magnetic resonance imaging, etc.) suffers from a certain hysteresis and constraint, and is therefore non-effective for early-stage or small-lesion lung cancer diagnosis (Wittekind and Neid, 2005). To meet the increasing demand for early detection of lung cancer and associated favorable prognosis, the national lung screening trial (NLST) proposed a chest low-dose computed tomography (LDCT) that could reduce the mortality rate by 20% (Shin et al., 2020). However, repeated LDCT would undoubtedly have radiation effects on the human body, leading to side effects on tissues, blood, and even the immune system. As a result, alternate high-safety diagnostic methods that can detect lung cancer at an early stage are urgently required.

Recently, liquid biopsy, a promising diagnostic method for the safe detection of cancer by capturing and recognizing cancer-related biomarkers in the body, has attracted a great deal of attention. Among all kinds of biomarkers, circulating tumor cell (CTCs) are deemed to be of primary importance, because they are the smallest units that contain the most complete information about tumor characteristics. To be specific, CTCs refer to tumor cells that are shed from the primary tumor or metastases, which are then released into body fluids. Usually, the majority of tumor-derived cells may reach circulation perish, while a tiny percentage of CTCs infiltrate distant organs and tissues, leading to tumor development and metastasis (Massagué and Obenauf, 2016). Therefore, the number of CTCs in peripheral blood was useful for monitoring cancer progression and informative for the assessment of therapeutic effects. However, the heterogeneity and extremely low concentration of these cells (approximately 1–10 CTCs per ml peripheral blood) make their isolation and detection arduous (Yee-de León et al., 2020).

To achieve simple isolation and analysis of CTCs from human peripheral blood, the methods commonly used in hospitals rely on two sorts of instruments. One is based on physical properties, such as size, density, deformability, and electric charges. In terms of size, the diameter of CTCs is about 10–20 μm, while that of white blood cells (WBCs) is in between of 7–12 μm. Typical method, such as ISET^®^ (Rarecells, Paris, French https://www.isetbyrarecells.com/), may classify CTCs and WBCs by using the threshold aperture size of 8 μm. In addition, ApoStream^®^ (Precision Medicine Group, Bethesda, US, https://www.precisionformedicine.com/) works significantly for small cells fractionation by using dielectric charge differences. The other takes advantage of biological properties, such as specific recognition between antibodies and antigens, i.e., the CellSearch^®^ system (Janssen Diagnostics, Beerse, Belgium, https://www.cellsearchctc.com/). Currently, the CellSearch^®^ system, the only technology approved by the U.S. food and drug administration (FDA), also a gold standard for CTCs isolation and recognition *via* immunostaining, is widely used due to its semiautomatic property (Alix-Panabières and Pantel, 2014). However, this approach relies on pathologists’ subjective assessment, skill level, and experience, which might decrease the accuracy of results.

To enable fully automated detection with high accuracy, Svensson and co-workers were the first who employed color features as input for a naive Bayes classifier, which successfully detected and counted CTCs in fluorescent images (Svensson et al., 2014). Unfortunately, this approach could not get rid of the limitations of complicated and changeable smear circumstances, uneven lighting, uneven staining, cell adherence, environmental contaminants, etc., resulting in a high false-positive rate and poor practicability (Svensson et al., 2015). Later, Mao and co-workers compared the classic machine learning approach to a recently developed deep learning method, so as to explore a new path for CTCs analysis (Mao et al., 2015). Their results revealed that the deep learning method outperformed machine learning by recognizing the specific CTCs mixed with red blood cells (at a mixing ratio of 1:10,000). Nowadays, deep learning methods, especially convolutional neural network (CNN), are booming in natural image analysis, indicating a great potential in medical image processing (Maier et al., 2019). Recently, He and co-workers utilized the python package Sobel operator for preprocessing the antibodies marked CTCs, bringing a significant recall of 90.3% (He et al., 2020). In pursuit of faster speed, Liu and co-workers enhanced the Faster-RCNN anchor point production step. Specifically, they diminished the imbalance between positive and negative data by altering and expanding the anchor point (Liu et al., 2019). Afterward, they changed the image size by using two ratios, namely 3:4 and 2:3, respectively, so as to avoid omitting information in the created feature map. Based on this, Li and co-workers then extended a deep multiscale residual network (DMRN) to enhance the performance (Li et al., 2019). However, the interpretability of medical images and the visualization of features have always been in its infancy (Wang et al., 2020), hence the importance of interpretatively automated identification of circulating tumor cells with high precision and rapid speed.

**FIGURE 1:**
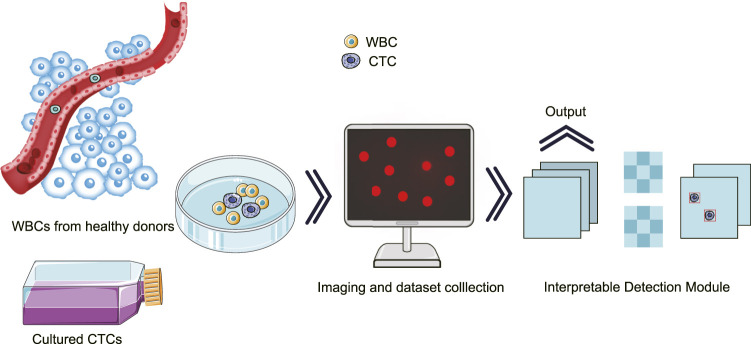
Schematic illustration for the interpretatively automated identification of CTCs in spiked samples, where CTCs were added in human peripheral blood containing WBCs.

To achieve the aforementioned goal of high-performance detection of CTCs in real samples, we innovatively optimized single-shot multi-box detector (SSD)-based neural network, in which attention and feature fusion were integrated (Nagrath et al., 2020; Magalhães et al., 2021). To note, the gradient-weighted class activation mapping (Grad-CAM) was added for interpretability function, and the t-distributed stochastic neighbor embedding (T-SNE) was used for visualization. Specifically, we first conducted blood processing and cell culture experiments for the acquisition of spiked samples. Then, the images of the spiked samples were taken for further processing. At the end, our results were graphically interpreted with high accuracy (Figure 1).

## 2 Materials and methods

### 2.1 Reagents

H1299, a non-small cell lung carcinoma cell line derived from the lymph node metastases of a 43-year-old Caucasian man, was chosen as model for this study. In addition, the Shanghai Ninth People’s Hospital contributed a total of sixty tubes of blood samples from healthy donors and lung cancer patients, respectively. To ensure the rigor and precision of the experiments, all blood samples were manually collected and processed within 48 h. According to the protocol approved by our Institutional Review Board (number ECSHU 2020–044), all procedures involving human participants were performed following the 1964 Helsinki declaration and its later amendments. Reagent supplies include but are not limited to phosphate buffered saline (PBS) (Thermo Fisher Scientific, Shanghai, China), paraformaldehyde fix solution (PFS) (Beyotime Biotechnology, Shanghai, China), and Roswell Park Memorial Institute (RPMI) 1,640 medium (SIGMA, Merck, Darmstadt, Germany), etc.

### 2.2 H1299 cell culture

H1299 was cultured in RMPI 1640 mediums supplemented with 10% fetal bovine serum (FBS) in a 75 cm^2^ flask at 37°C with 5% CO_2_ and 100% humidity.

### 2.3 WBCs extraction

First of all, 6 ml erythrocyte lysate was added into 2 ml blood for 15 min incubation in an ice bath. After centrifuging at 2000 rpm for 10 min, the majority of red blood cells were removed, leading to human peripheral blood containing WBCs. At the end, the remaining WBCs were obtained by rinsing with D’HANKS solution and stored in RMPI 1640 for use within 24 h.

### 2.4 Data acquisition

For the creation of dataset, the cultured H1299 was digested with trypsin, then collected and fixed with 4% PFS. Afterward, the cells were stained with 4′, 6-Diamidino-2′-phenylindole (DAPI) for 30 min, and then suspended in PBS containing 10% Tween. Using a cell counting plate, H1299 cells were mixed with the obtained WBCs at a ratio of 1:10,000 and a concentration of 500 H1299 cells/mL.

In this section, the confocal microscope (Zeiss LSM 710) was employed for the sample imaging. The magnification was set to ×20, and the pixel was set to 1,024 × 1,024 pixel^2^ in 16 bits, with absorption at 359 nm and emission at 461 nm. At the end, 80 raw images were taken for further processing.

### 2.5 Data preprocessing

By randomly extraction from the middle 850 × 850 pixel^2^ area of the raw images, a total of 2,400 images at 300 × 300 pixel^2^ with similar amount of cells was manually selected to constitute Dataset I. For Dataset I, the gamma transformation was performed for brightness reducing, and histogram normalization was used to guarantee characteristic identification, as defined in Equation 1 and (2). Here, r and c are the row and column, respectively. I is the input image, and O is the output image. Besides, (.)_max_ and (.)_min_ are represented as the largest and smallest grayscale values in the image. *γ* is considered as the parameter that need to be provided and refined.

$$O\left(r,c\right)=I{\left(r,c\right)}^{\gamma }$$ (1)

$$O\left(r,c\right)=\frac{O_{\max}-O_{\min}}{I_{\max}-I_{\min}}\left(I\left(r,c\right)-I_{\min}\right)+O_{\min}$$ (2)

Toward the 80 images correlated XML files, the image enhancement was conducted by translating 40/60 pixels on the x/y axis, scaling to 50%–70%, flipping and mirroring horizontally. According to the above-mentioned biological gold standard, the labeling of the H1299 region was accomplished by using the Labelme toolkit. Followed, a Dataset II constituted by 1,000 single-cell images were generated.

### 2.6 Automated identification

#### 2.6.1 Optimized SSD

VGG16 was selected as the feature extraction network backbone, so as to facilitate the multi-scale feature extraction for tiny objects identification. At points in the image, each layer was heavily and evenly sampled; various scales and aspect ratios were used for accurate identification. To eliminate the duplicate detection, a post-processing strategy, namely non-maximum suppression (NMS), was applied by calculating the intersection ratio of the predicted frame and the ground-truth frame.

#### 2.6.2 Attention mechanism and feature fusion

**FIGURE 2:**
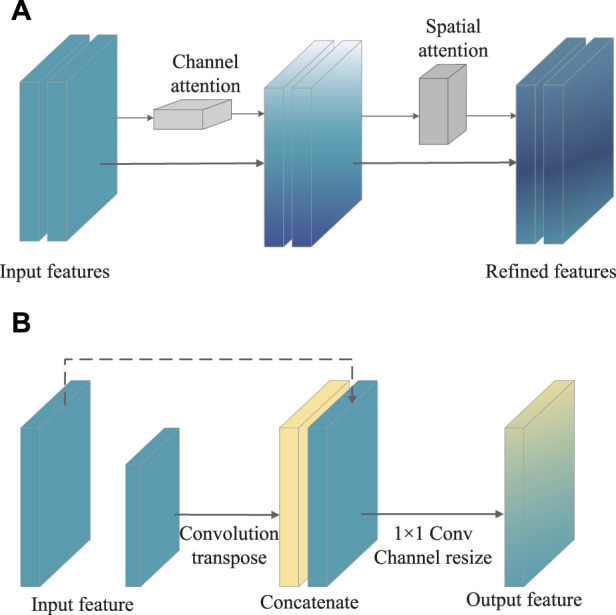
Schematic illustration of CBAM structure **(A)** and feature fusion module **(B)**.

To achieve the best combination of speed and precision, the upgraded SSD was proposed by adding with attention mechanism and feature fusion modules after the multi-scale feature extraction from the SSD. Attention allows people to rapidly focus on the most useful part of the scene. Similarly, in vision tasks such as image classification, segmentation and detection, the attention mechanism enables the neural network to focus on local important details instead of global situation, which greatly enhances the performance of the network. As shown in Figure 2A, the convolutional block attention module (CBAM) that combined with the spatial and channel attention modules was added to the first four layers of SSD (Woo et al., 2018). For channel attention, the features extracted from any type of convolutional neural network were processed and, *via* max pooling and average pooling, then channel-wise responses were obtained. Through a shared network, the descriptors were extracted, which were ultimately regarded as the final weights on the original input features. After processing *via* the channel attention module, the channel-wise weighting features were generated, which served as the input to the spatial attention module. Complementary to the channel of attention, spatial attention focuses on the informative parts. The descriptors were generated by applying average and max pooling along with the channel axis and the spatial attention map.

At the same time, both early and late fusion were adopted in the SSD (Figure 2B). First, the early fusion fused the features of multiple layers, and then trained the predictor on the fused features. Afterward, the late fusion generated feature layers as detection results. More in details, the high-level feature was sized-recovered by exploiting convolution transpose, and then concatenated to the processed feature. Ultimately, a 1 × 1 Conv that carried more texture and fundamental messages might yield channel resizing for feature output.

#### 2.6.3 T-SNE and Grad-CAM

**FIGURE 3:**
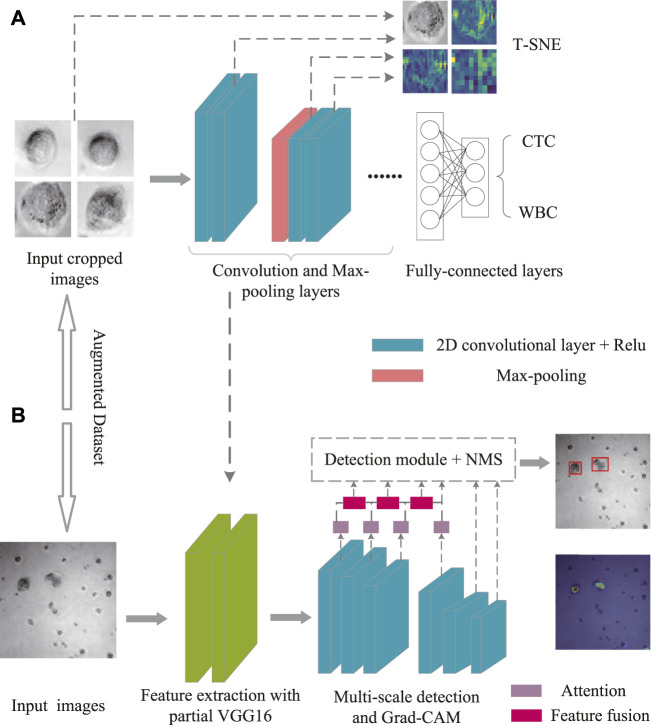
Schematic illustration of T-SNE for Dataset II treatment **(A)** and Grad-CAM for Dataset I analysis **(B)**.

For data visualization, T-SNE offers an excellent data dimension reduction approach by converting the data points into probabilities (van der Maaten and Hinton, 2008). Specifically, the Gaussian joint probability represents similarity in the original space, whereas the “Student’s t distribution” represents similarity in the embedded space. The Kullback-Leibler (KL) divergence of the joint probability of the original space and the embedded space was employed to assess the quality of the visualization. Figure 3A depicts the construction of the VGG16 classification architecture, which was performed to differentiate between CTC and WBC. Various feature maps were then retrieved from various levels. After that, the feature maps were treated *via* T-SNE.

Neural networks pay attention to the portions to be recognized, herein Grad-CAM (Selvaraju et al., 2017) was used for effective detection (Figure 3B). Specifically, the heat maps were utilized to highlight the main elements of the image for determining a certain kind. Meanwhile, Grad-CAM could provide the class activation map with no need of retraining or changing the structure of model. To note, the first three of the multi-level features were fused with the subsequent features through the CBAM module, and the expected fusion results were collected for the detection, while the fourth was only used as an auxiliary fusion module. As shown in Eq. 3, the visual interpretation for a specific object detection is defined as $L_{ij}^{c}$ , where (i, j) is the spatial location of the specific class c.

$$L_{ij}^{c}=\underset{k}{\sum }w_{k}^{c}\cdot A_{ij}^{k}$$ (3)

**FIGURE 4:**
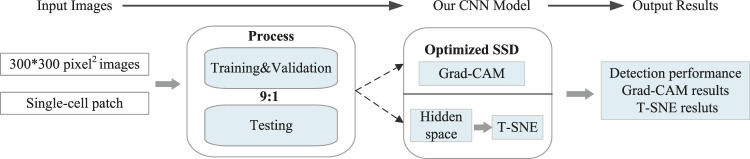
Overview of the generated CNN model for automated detection.

The weight $w_{k}^{c}$ of class c and the feature map *A* ^*k*^ are defined in Eq. 4, where *Y* ^*c*^ is the final detection score of class c. Moreover, Z is a constant that represents the number of pixels in the activation map. To be specific, our CNN model was generated by the training of two data sets, followed by the 5 fold Cross Validation (the ratio of training set and test set is 9:1). It is worth mentioning that in our model, hidden space is the space where the results of convolution of each layer is located, while T-SNE is to visualize the information in hidden space. Usually, the relevant detection results can be automatically output *via* this model (Figure 4).

$$w_{k}^{c}=\frac{1}{Z}\underset{i}{\sum }\underset{j}{\sum }\frac{\partial Y^{c}}{\partial A_{ij}^{k}}$$ (4)

#### 2.6.4 Evaluation metrics

In order to assess the overall model’s level of performance and its capacity for discrimination, several different assessment criteria were used, such as precision, recall, average precision (AP) and speed value (frames per second, FPS). For a short but important introduction, the intersection over Union (IOU) is an important concept employed in the non-maximum suppression (NMS) for the subsequent computation of AP. Usually, IOU is to assess the target’s capacity to identify the expected frame. With the projected frame as a starting point, the IOU can be calculated as ratios with the genuine bounding box. For example, if the threshold for the IOU is set at 0.5, then the true positive (TP) indicates the ratio of which the IOU is higher than 0.5. The number of ground truths that cannot be detected is denoted by the variable false negative (FN). On the other hand, the number of detection boxes for false positive (FP) is equal to the number of detection boxes that are present when the IOU is less than or equal to 0.5.

$$Precision=\frac{TP}{TP+FP}$$ (5)

$$Recall=\frac{TP}{TP+FN}$$ (6)

Precision and Recall were calculated according to the Equation 5 and (6), respectively. Commonly, the precision decreases when the recall is high. In this work, the precision-recall (PR) curve was plotted, then AP value was obtained by calculating the area under PR.

## 3 Results and discussion

### 3.1 Data preprocessing and the setup

**FIGURE 5:**
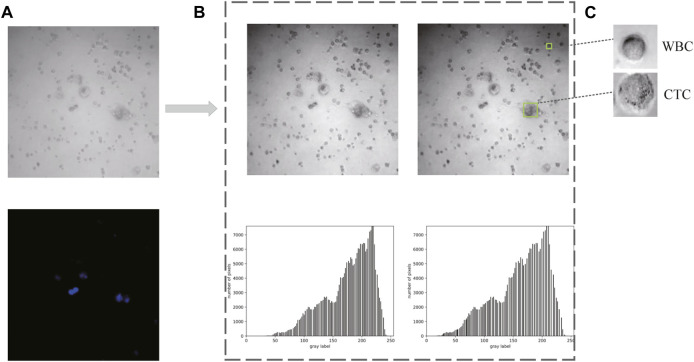
Data acquisition and preprocessing. **(A)** Raw images obtained from confocal microscope. **(B)** Preprocessed images and the corresponding histograms. **(C)** Cropped images with single cell for dataset constitution.

As shown in Figure 5A, 80 bright field images and their corresponding DAPI-stained images were ultimately collected in the first place. Afterward, two methods (i.e., gamma transformation and histogram normalization) were introduced here to guarantee identical characteristics of each image in middle 850 × 850 pixel^2^, such as sharpness, contrast, and brightness (Figure 5B). Since the output size of these raw images was 1,024 × 1,024 pixel^2^, leading to the size of each cell often less than the relative ratio of 0.32. Thus, a total of 2,400 images (extracted from the middle 850 × 850 pixel^2^, with resolution at 300 × 300 pixel^2^) with similar number of cells were collected as Dataset I. Besides, combined with the corresponding DAPI-stained labels, 1,000 images with single-cell were generated to form the Dataset II. (Figure 5C).

All experiments were conducted on a server with Intel(R) Xeon(R) Silver 4216 CPU @ 2.10 GHz, equipped with two NVIDIA GeForce GTX 3090 GPU on an Ubuntu 18.04 operating system. And there were a total of 100 iterations throughout the training phase. In addition, trained model parameters in the ImageNet were also used for transfer learning, in which 50 epochs were equally established for the freezing and unfreezing phase. During the freezing-phase training, the model’s backbone was frozen, and the network for feature extraction did not change while in the unfreezing phase of training, all model parameters were updated. Furthermore, the ratio of training, testing, and validation data was set to 0.8:0.1:0.1 using Adam as optimizer due to its good properties. The exponential decay rate of the first-order moment estimates (beta1) was set to be 0.9, and the second-moment estimates (beta2) to be 0.999. Additionally, the initial learning rate was set to be 6e-4 so as to exploit cosine decay with the rate of weight loss at 5e-4.

Eq. 7 describes the loss function of the upgraded SSD, which includes the regression loss for the predictions of all positive-label boxes, the cross-entropy loss of the prediction results for all positive label categories, and the cross-entropy loss of the prediction result of a certain type of negative label. Using the loss function, negative samples during training were restricted by changing the ratio of negative samples to positive samples from 3 to 1.2. At the same time, early stopping was used to avoid over-training and wasting resources. Furthermore, owing to presence of abundant small objects in the image, the anchor size was adjusted to [21, 45, 99, 153, 207, 261, 315].

$$L_{\mathrm{total}}=L_{\mathrm{reg}}^{\mathrm{pos}}+L_{\mathrm{clas}}^{\mathrm{pos}}+L_{\mathrm{clas}}^{\mathrm{neg}}$$ (7)

### 3.2 Performance comparison for upgraded SSD

Usually, the Faster-RCNN performs target detection *via* two stages (Ren et al., 2015). One is ROI screening, which plays an important role in distinguishing a large number of foreground and background targets. The other is the classification of targets and the regression of candidate boxes. Faster-RCNN boosts the addition of region proposal networks (RPN), which assists in extracting features and improving the detection speed. However, while extracting features, the RPN network only employs the findings of the previous layer, so the detection ability for tiny targets is suboptimal. Unlike the two-stage detector, you look only once (YOLO) integrates box regression and categorical determination (Redmon and Farhadi, 2018). It is currently the fastest one-stage detector, which applies a feature pyramid network to perform multiscale detection, thus is beneficial for detecting small targets. Yet, its detection performance is not promising due to its high error ratio. Another representative one-stage object detection algorithm, i.e., SSD that carries out object categorization and prediction frame regression simultaneously, has shown a great prospective (Liu et al., 2016). Moreover, RetinaNet (Lin et al., 2017) and EfficientDet (Tan et al., 2019) are well-known for being comparable in accuracy to the two-stage detectors. Table 1 presents the performance of Faster-RCNN, YOLO, RetinaNet, EfficientDet and SSD. Though the prediction precision of Faster-RCNN was the best when the IOU was 0.5, somewhat unsatisfactory. Moreover, the RetinaNet model received the highest recall rate among the five models, the speed was the lowest. In view of this, we speculate that adding the attention mechanism and feature fusion function in the RetinaNet model or Faster-RCNN model will reduce the speed even more. Meanwhile, the EfficientDet model fared poorly in terms of recall rate and AP, as well as YOLO model. Therefore, the comparative experiments show that our upgraded SSD is the algorithm model with the greatest application potential in the case of comprehensive consideration of AP value and speed.

**TABLE 1:** Detection performance comparison of five models.

	Recall	Precision	AP	FPS
Faster-RCNN	0.869	0.981	0.941	52.9
YOLO	0.828	0.920	0.911	123.5
RetinaNet	0.898	0.978	0.929	45.6
EfficientDet	0.784	0.969	0.899	71.1
SSD	0.859	0.948	0.955	111.9

### 3.3 SSD development

**FIGURE 6:**
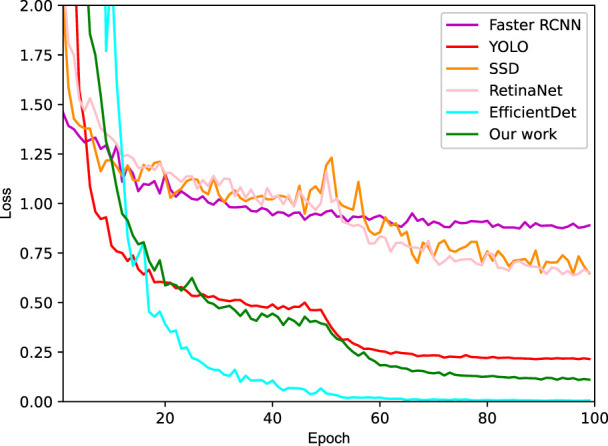
Loss curves of four different methods.

For performance improvement, the attention mechanism which can include contextual semantic information and reduce the interference of low-level information, was integrated into our optimal SSD. Moreover, low-level features contain more location and detail information, but have less semantics and more noise due to fewer convolutions. On the contrary, the high-level features have stronger semantic information, yet the ability to perceive details is less impressive. Thus, Feature fusion module was applied in our SSD, so as to combine the two features for performance improvement. After 100 epochs of training, the loss tended to converge, which would benefit the test sample generalization. And a comparison of four methods was conducted as shown in Figure 6, indicating that our SSD + Attention + Feature fusion method is comparable to YOLO.

Specifically, ablation experiments were carried out as summarized in Table 2. According to this table, no matter which improvement strategy was exploited, the detection performance was boosted compared with no addition. And significantly, the effect of only adding attention enhancement outperformed that of only feature fusion. In the case of SSD integrated with the attention mechanism and feature fusion, and after balancing the gap between positive and negative samples, the recall was boosted to 92.2%, and the AP reached 97.9%. Importantly, the main contributor to performance improvement was attention function. Although the FPS of our work has dropped to 72.9 compared to the SSD, it was also sufficient for real-time requirements considering the human detecting threshold for video frame changes of 30 FPS.

**TABLE 2:** Ablation experiments.

	Recall	AP	FPS
SSD	0.859	0.956	111.9
SSD + Attention	0.891	0.970	76.3
SSD + Feature fusion	0.903	0.969	100.7
SSD + Attention + Feature fusion	0.922	0.979	72.9

### 3.4 Interpretability and visualization

**FIGURE 7:**
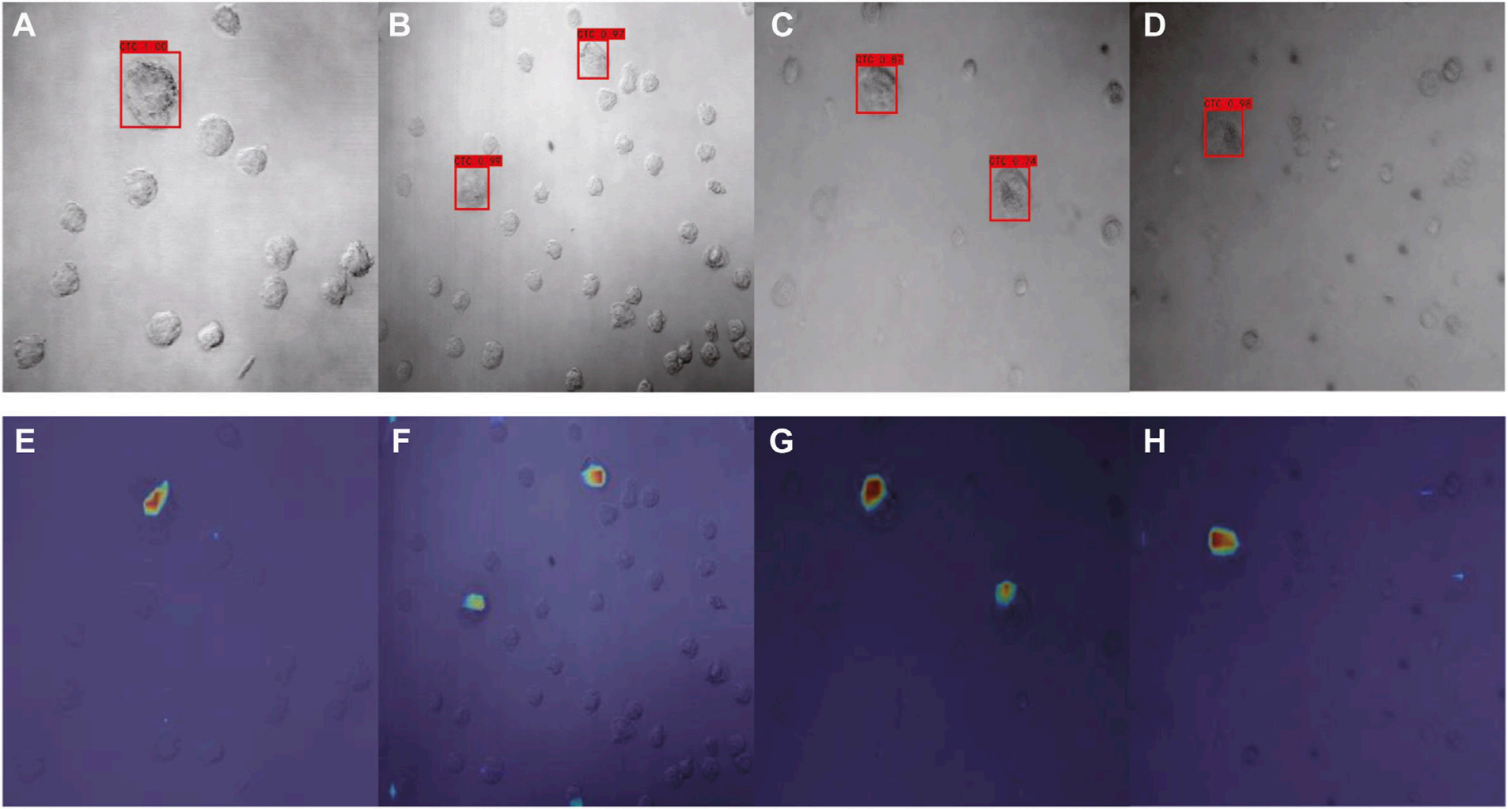
Detection results of optimized SSD **(A–D)** and the corresponding interpretation obtained by Grad-CAM **(E–H)** for Dataset I.

Using our optimized SSD, the category name and the confidence value of the category was generated for each image *via* the corresponding confidence convolutional layers of each block (Figures 7A–D). For model interpretability, the output mapping of different layers of the network was then combined with the Grad-CAM method. There were a total of six layers of feature map visualization results in our work. However, the later layers had higher scales, so the weighted sum strategy was implemented, in which the weight of low-level semantic information was higher. Figures 7E–H depict the corresponding model interpretation obtained by utilizing Grad-CAM. This heat map shows the sensitivity of the network to the objects after training. Specifically, the red area in the figure indicates that this area contributed the most to the determination of CTC.

**FIGURE 8:**
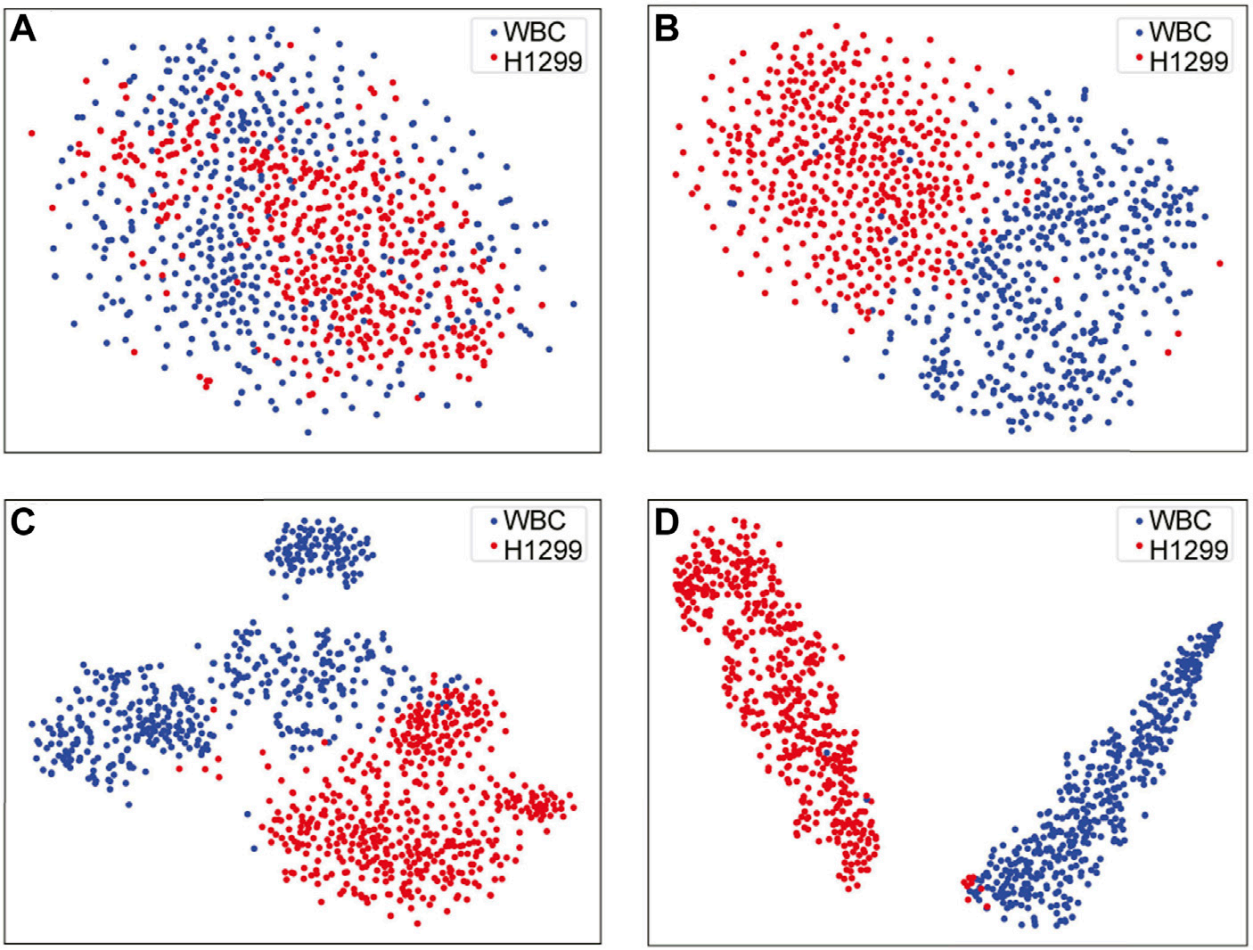
Dataset II visualization *via* T-SNE **(A–D)**.

For feature visualizing, T-SNE reduced the dimension of high-dimensional image data, and the outputs of different blocks in our work were taken as the input data for the dimensionality reduction, where the distribution of data and features were constructed in t-space. As shown in Figure 8, where result A was the original data distribution that directly obtained from the input image of VGG16. Followed, the results B, C, and D are visualized distributions after passing through the fifth and seventh convolutional layers and the last fully connected layer of VGG16, respectively. The above results demonstrate the classification ability of VGG16 to a certain extent, were afterwards generalized to the SSD skeleton of our work, indicating that the visualization function was of great significance in evaluating the obtained detection results.

## 4 Conclusion

In summary, we successfully developed an innovative method for the automated identification of CTCs in spiked samples, which also works superior with respect to other algorithm models on other datasets (Supplementary Table S1). For the first time, a new SSD with attention and feature fusion was constructed, utilizing VGG16 trained by our own dataset. To note, the application of Grad-CAM and T-SNE made the output of our algorithm visual, interpretable and therefore more reliable. It turns out that our AP value has reached 97.9%, which was the most significant performance with respect to other algorithms. Thus, our work opens a new perspective the invented algorithm for various cancer detection and more reliable diagnostic output.

## Data Availability

The raw data supporting the conclusions of this article will be made available by the authors, without undue reservation.
