# Supplementary material for: Interpretatively automated identification of circulating tumor cells from human peripheral blood with high performance
Source: Front Bioeng Biotechnol. 2023 Feb 9;11:1013107. doi: 10.3389/fbioe.2023.1013107 (PMC9947588; doi:10.3389/fbioe.2023.1013107)
Supplement: Supplementary file 5 [file DataSheet1.PDF]

Supporting Information

Interpretatively Automated Identification of Circulating Tumor Cells from Human Peripheral Blood with High Performance

Xiaolei Li[1], Mingcan Chen[1], Jingjing Xu[1]*, Dihang Wu[1], Mengxue Ye[1], Chi Wang[2]*, Wanyu Liu[1]*

[1] Sino-European School of Technology of Shanghai University, Shanghai University, CN-200444 Shanghai, P. R. China

[2] School of Mechatronic Engineering and Automation, Shanghai University, CN-200444 Shanghai, P. R. China

Correspondence*: jingjing xu@shu.edu.cn, wangchi@shu.edu.cn, liuwanyu@shu.edu.cn.

Performance comparison on VOC2007+2012 dataset

For a more convincing comparison, the performance of various algorithm models, such as Faster-RCNN, YOLO, RetinaNet, EfficientDet, SSD, our CNN model, was verified on the public VOC2007+2012 dataset. The results show that Faster-RCNN, YOLO and EfficientDet did not perform very well on mRecall or mPrecision. On the other hand, our CNN model outperformed RetinaNet and SSD in terms of mAP, indicating a great potential for clinical uses.

Table S1. Detection performance comparison of various models.

Algorithm model	mRecall	mPrecision	mAP
Faster-RCNN	0.883	0.569↓	0.848
YOLO	0.669↓	0.903	0.824
RetinaNet	0.789	0.808	0.821
EfficientDet	0.663↓	0.894	0.752
SSD	0.825	0.923	0.818
Our model	0.887	0.906	0.828
